# Circulatory and prostatic tissue lipidomic profiles shifts after high-dose atorvastatin use in men with prostate cancer

**DOI:** 10.1038/s41598-020-68868-5

**Published:** 2020-07-21

**Authors:** Paavo Raittinen, Kati Niemistö, Erika Pennanen, Heimo Syvälä, Seppo Auriola, Jarno Riikonen, Terho Lehtimäki, Pauliina Ilmonen, Teemu Murtola

**Affiliations:** 1 Department of Mathematics and Systems Analysis, Aalto University School of Science, Helsinki, Finland; 2 Tampere University, Tampere, Finland; 3 School of Pharmacy, University of Eastern Finland, Kuopio, Finland; 4 Faculty of Medicine and Health Technology, Tampere University and Tays Cancer Center, Tampere University Hospital, Tampere, Finland; 5 School of Pharmacy, Faculty of Health Sciences, University of Eastern Finland, Kuopio, Finland; 6 Tays Cancer Center, Tampere University Hospital, Tampere, Finland; 7 Department of Clinical Chemistry, Faculty of Medicine and Health Technology, Fimlab Laboratories and Finnish Cardiovascular Research Center-Tampere, Tampere University, Tampere, Finland

**Keywords:** Metabolomics, Lipidomics, Cancer therapy, Cancer metabolism, Prostate cancer

## Abstract

Prostate cancer patients using cholesterol-lowering statins have 30% lower risk of prostate cancer death compared to non-users. The effect is attributed to the inhibition of the mevalonate pathway in prostate cancer cells. Moreover, statin use causes lipoprotein metabolism changes in the serum. Statin effect on serum or intraprostatic lipidome profiles in prostate cancer patients has not been explored. We studied changes in the serum metabolomic and prostatic tissue lipidome after high-dose 80 mg atorvastatin intervention to expose biological mechanisms causing the observed survival benefit. Our randomized, double-blind, placebo-controlled clinical trial consisted of 103 Finnish men with prostate cancer. We observed clear difference in post-intervention serum lipoprotein lipid profiles between the study arms (median classification error 11.7%). The atorvastatin effect on intraprostatic lipid profile was not as clear (median classification error 44.7%), although slightly differing lipid profiles by treatment arm was observed, which became more pronounced in men who used atorvastatin above the median of 27 days (statin group median classification error 27.2%). Atorvastatin lowers lipids important for adaptation for hypoxic microenvironment in the prostate suggesting that prostate cancer cell survival benefit associated with statin use might be mediated by both, local and systemic, lipidomic/metabolomic profile changes.

## Introduction

Prostate cancer (PrCa) is the most common cancer and causes the second most cancer deaths among men in western countries^1^. Known risk factors for PrCa are increasing age, screening behaviour, race, and family history of prostate cancer^2,3^.

Increased fat intake and especially hypercholesterolemia has been associated with increasing risk of advanced/aggressive PrCa, but not with overall PrCa risk^4–6^. Cholesterol is a precursor to androgens, which are necessary in the development and growth of the prostate. Moreover, prostate cancer cells have been observed to reprogram their lipid metabolism during hypoxic conditions as a pathway to proliferate^7^.

Lipids have important biological functions that include storing energy, cell signaling and acting as structural components in the cell membranes. Fatty acid and cholesterol biosynthesis are dysregulated in PrCa cells^8^. PrCa cells also have increased intake of fatty acids^9^. Lipid metabolism has been found to have a crucial role in tumor growth and metabolism^10^. Few studies have explored serum lipidome in PrCa patients aiming to find new biomarkers for PrCa diagnostics. In these studies differences were found in a few serum lipids between PrCa patients and healthy controls^11,12^. Recently Brzozowski et al. demonstrated differing lipidome profiles in extracellular vesicles (EVs) derived from prostate cancer cell lines compared to non-cancerous cells^13^.

Use of cholesterol-lowering statins has been linked to better prognosis and lowered risk of metastatic PrCa^14,15^. It is still unsure to what extent the lowered risk for metastatic PrCa in statin users result from (1) systemic changes in lipoprotein metabolism and cholesterol levels or (2) local changes in the prostate tissue. There are no published studies on how statin use affects serum lipoprotein metabolism or lipidome in PrCa patients and whether statin use associates with intraprostatic lipidome shift.

In this study, we examine the association between atorvastatin use and lipidomic changes among men with PrCa. In particular, we studied both, serum lipoprotein lipid and fatty acid metabolism and intraprostatic lipidome wide shift. Our cohort consists of 103 Finnish men with PrCa in the context of randomized double-blind, placebo-controlled clinical trial^16^.

## Materials and methods

### Study population

The study population included men (n = 103, aged 49–75 years, median 65) with histologically confirmed previously untreated PrCa that were scheduled for robot-assisted radical prostatectomy at Tampere University hospital, Tampere, Finland^16^ (Table 1). Study was registered by clinicaltrials (01/04/2013, identifier: NCT01821404) and EudraCT (21/07/2013, registration number: 2011-005438-20), and was approved by the ethics committee of Pirkanmaa Hospital District (decision number ETL R03230). The study was performed in accordance of Declaration of Helsinki and good clinical practice. Study exclusion criteria were previous oncological treatment for any malignancy, previous usage of any statin or 5α-reductase inhibitor within a year from the recruitment. Patients with clinically significant liver or kidney insufficiency were excluded, as well as patients with ongoing use of drugs interacting with statins, and men who had history of adverse effects from cholesterol-lowering treatment. There are some differences in baseline smoking, hypertension, and diabetes between the treatment arms (Table 1). The possible impact of these baseline imbalances were evaluated by testing the statistical significance of the median lipid level difference by smoking, hypertension, and diabetes using Wilcoxon rank-sum test^17^. An informed consent was obtained from all the participants in this study. The study protocol, including randomization procedure, has been previously described in detail^16^.

**Table 1:** Participant characteristic summary table per study arm.

Participant characteristics	Placebo (48 men)	Atorvastatin (55 men)
Age, median (IQR)	64.5 (10)	65 (9)
BMI, median, (IQR)	26.6 (4.1)	26.4 (4.9)
Smoking, n (%)	8 (17)	13 (23.6)
Hypertension, n (%)	16 (34)	21 (38.2)
Diabetes mellitus, n (%)	6 (12.8)	4 (7.3)
Intervention duration, median (range)	27 (13–76)	28 (10–114)
**Pathological Gleason grade, n (%)**		
ISUP Gleason grade ≤ 2	37 (78.7)	42 (76.3)
ISUP Gleason grade ≥ 3	10 (21.2)	13 (23.6)
**Pathological T-stage, n (%)**		
T2b or lower	1 (2.1)	5 (9.0)
T2c or higher	46 (97.9)	50 (91)

*BMI * Body Mass Index; *IQR*   interquartile range, *ISUP* International Society of Urological Pathology.

### Intervention with high-dose atorvastatin

The study population was randomized 1:1 to receive either 80 mg atorvastatin or placebo from the recruitment until radical prostatectomy. The participants and the study physicians were blinded to the treatment allocation by identical outlook and weight of the study drugs containing atorvastatin or placebo. The drug capsules were randomized and blinded at the drug manufacturing site and each pill box was assigned with an ID number ensuring the allocation concealment. Each patient was given an ID number and a corresponding pill box containing the drugs. Compliance was monitored by calculating leftover capsules at the time of surgery and comparing it to the number of doses participants should have taken. Overall compliance was 96%^16^. After the follow-up, the patients in the placebo arm were queried about any post randomization statin use and no one reported using statins. The intervention time varied according to waiting time to radical prostatectomy. No minimum exposure time could have been set as the ethics committee of Pirkanmaa Hospital District required that cancer treatment cannot be delayed due to the study. The observed median statin use time prior to the surgery was 27 days.

### Nuclear magnetic resonance (NMR) based analysis of plasma lipid and lipoprotein lipid profiles

Blood samples for measurement of serum metabolites were taken at recruitment and again before the surgery^16^. A high-throughput targeted nuclear magnetic resonance (NMR) metabolomics platform (Nightingale Health Ltd, Helsinki, Finland) was used to quantify 204 serum lipoprotein lipid and fatty acid related indices (hereafter named as serum lipidome) and 20 other type of metabolites as absolute concentrations of each metabolic measure or as ratios (see Supplementary file 1). After internal data validation, 212 serum metabolites before and 190 serum metabolites after the intervention were consistently defined for all 103 participants, which were used in the analysis. This metabolite panel captures biomarkers from multiple metabolic pathways, including fatty acids and detailed lipoprotein lipid profiles, covering triacylglycerol, total cholesterol, non-esterified cholesterol, esterified cholesterol and phospholipids within 14 different diameter lipoprotein subclasses. The NMR platform in experimentation use is described in detail elsewhere^18,19^.

### Lipidomic analysis of intraprostatic tissues using liquid chromatography high-resolution mass spectrometry

**Figure 1:**
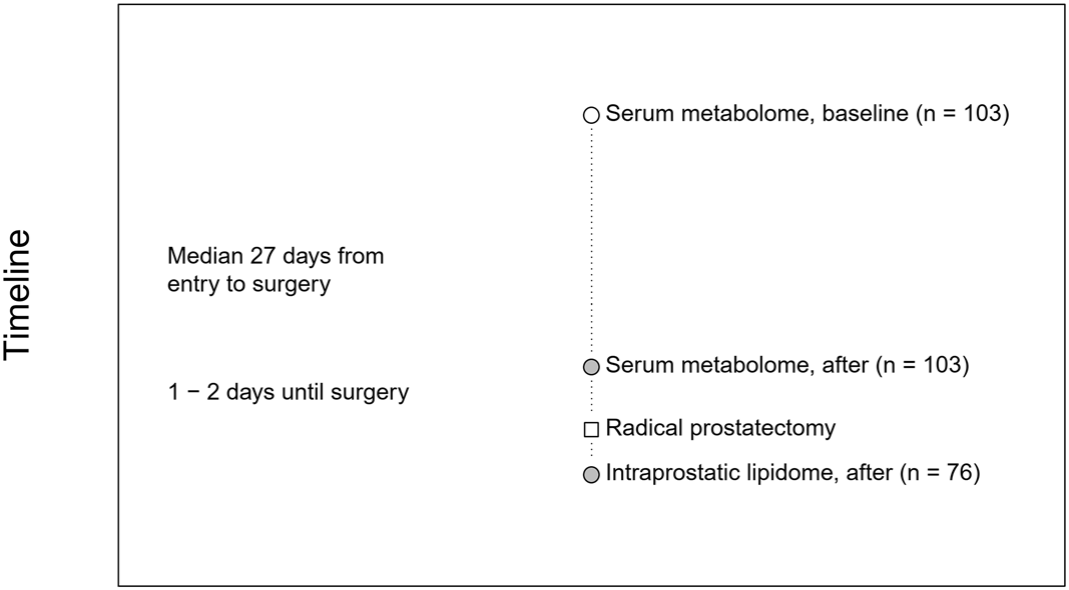
Schematic representation of the serum lipid and lipoprotein lipid and intraprostatic tissue lipidome measurement time-points. The serum lipid and lipoprotein lipid content was obtained at two different time-points, whereas the intraprostatic lipidome is obtained only once after the surgery.

A fresh cut from macroscopically cancer-free tissue was obtained immediately after the surgery and stored into liquid nitrogen for lipidome profiling^16^. The intraprostatic lipidome was measured using liquid chromatography high-resolution mass spectrometry (LC–MS/MS). The detailed methodology and reagents, as well as the process of lipid identification are described in Supplementary file 2. The intraprostatic lipidome covering 522 lipids was consistently determined for 76 patients, 36 taking placebo and 40 using high-dose atorvastatin treatment (Supplementary file 1). An illustration of the timeline when a corresponding lipoprotein lipid and lipidomic profiles were analyzed is presented in Fig. 1.

### Statistical analysis of lipid metabolome and lipidome profiles

The association between statin or placebo use and lipidome shift was modelled using supervised random forest classification (RFC) algorithm^20^ using R version × 64 3.5.1 and randomForest package version 4.6–14. Since the study arms are known a priori and there is no significant class imbalance the association can be modelled as a supervised classification task. Furthermore, RFC is ample at handling high-dimensional data in an exploratory study setting and provides well interpretable results. The LC–MS/MS LC–MS/MS based prostatic tissue lipidomic or NMR-based serum lipid and lipoprotein lipid profiles were used as classifiers whereas the study arm as the response to expose any association between atorvastatin intervention and individual lipids.

The serum lipid and lipoprotein lipid shift due to atorvastatin intervention was investigated using baseline serum profile (incl. 212 metabolite measures) and post intervention serum metabolic profile (190 metabolite measures) as RFC classifiers. The 522 intraprostatic lipids were reduced to a smaller subset of lipids as the expected small proportion of relevant intraprostatic lipids to non-relevant intraprostatic lipids can make RFC inefficient due to minimal change of relevant lipids being selected as classifiers in each RFC tree. The intraprostatic lipid subset was obtained by conducting Wilcoxon rank-sum test between the study arms for each lipid using unadjusted significance level alpha 0.05. The reduced intraprostatic lipidome profile used in the analysis contained 19 lipids which were selected on the basis that the corresponding *p*-values were less than 0.05. The impact of baseline differences in smoking, hypertension, and diabetes status to the model performance was evaluated by including them as classifiers in separate models.

As a planned post hoc analysis, the effect of Gleason grade and atorvastatin use time (days) was investigated by forming relevant sub-populations. The atorvastatin exposure time sub-populations were formed by dividing the cohort into hi-expose (men who used atorvastatin more than 27 days i.e., above group median) and lo-expose (men who used atorvastatin less than 27 days, less than group median) groups and making separate RFC models using the reduced intraprostatic lipidome as classifier. The effect of cancer aggressiveness to the intraprostatic lipidome shift due to atorvastatin use was studied by making separate RFC models for men with Gleason score 7 or lower, and Gleason score 7 or higher. The men with Gleason score 7 are included in both models as otherwise the groups would be too small for analysis.

The accuracy of the RFC prediction was achieved by comparing the predicted classes to the a priori known study arms, to obtain average error (out-of-bag-error in RFC terms) and *per* class errors. Each RFC model was ran 1,000 times to obtain an empirical median classification error estimate. Furthermore, to evaluate how much the RFC algorithm causes deviation due to its inherent random sampling, a 95% empirical confidence interval estimate was obtained by finding 97.5th and 2.5th percentiles from the ordered set of 1,000 classification errors (percentile method). Low deviation and low median classification error together are a sign of systematic structure in the data, therefore being an indication about the association between the classifiers and the response. Since we obtain median classification errors and confidence interval estimates, the results from each of the model can be compared more reliably.

Furthermore, the RFC algorithm measures the variable importance, i.e., how well a single classifier (lipid) is making a correct distinction between the classes (study arms)^21^. We obtained the most frequent top-performing metabolites in the serum profile RFC model and visualized the scaled and centered (mean 0, variance 1) lipid distributions *per* study arm as boxplots for both, serum profile before and after the intervention. In detail, we recorded the top five predictors from all 1,000 repetitions per model according to Gini split index and obtained unique set of predictors out of the 5 × 1,000 predictors. A low number of unique predictors is an indication of a stable model whereas high number of unique predictors indicates unstable model. The limitation enables descriptive and detailed view on how the lipidomic profile location and dispersion differs between the study arms in the best discriminating metabolites. In addition, the RFC algorithm yields an *N* times *N* proximity matrix, where *N* is the number of observations, which we used to visualize the difference between the study arms after multi-dimensional scaling of the proximity matrix^21,22^. If two points (one point *per* patient) are close to each other in a proximity plot, they demonstrate similarity to each other based on the classifiers used, whereas two points far from each other are considered as dissimilar. Furthermore, if proximity plots show clear clusters, it is an indication of good RFC classification accuracy.

Additionally, the significance of the median lipid level difference was studied using Wilcoxon rank-sum test^17^. Furthermore, the *p*-values were adjusted by using Benjamini–Hochberg method^23^.

## Results

**Figure 2:**
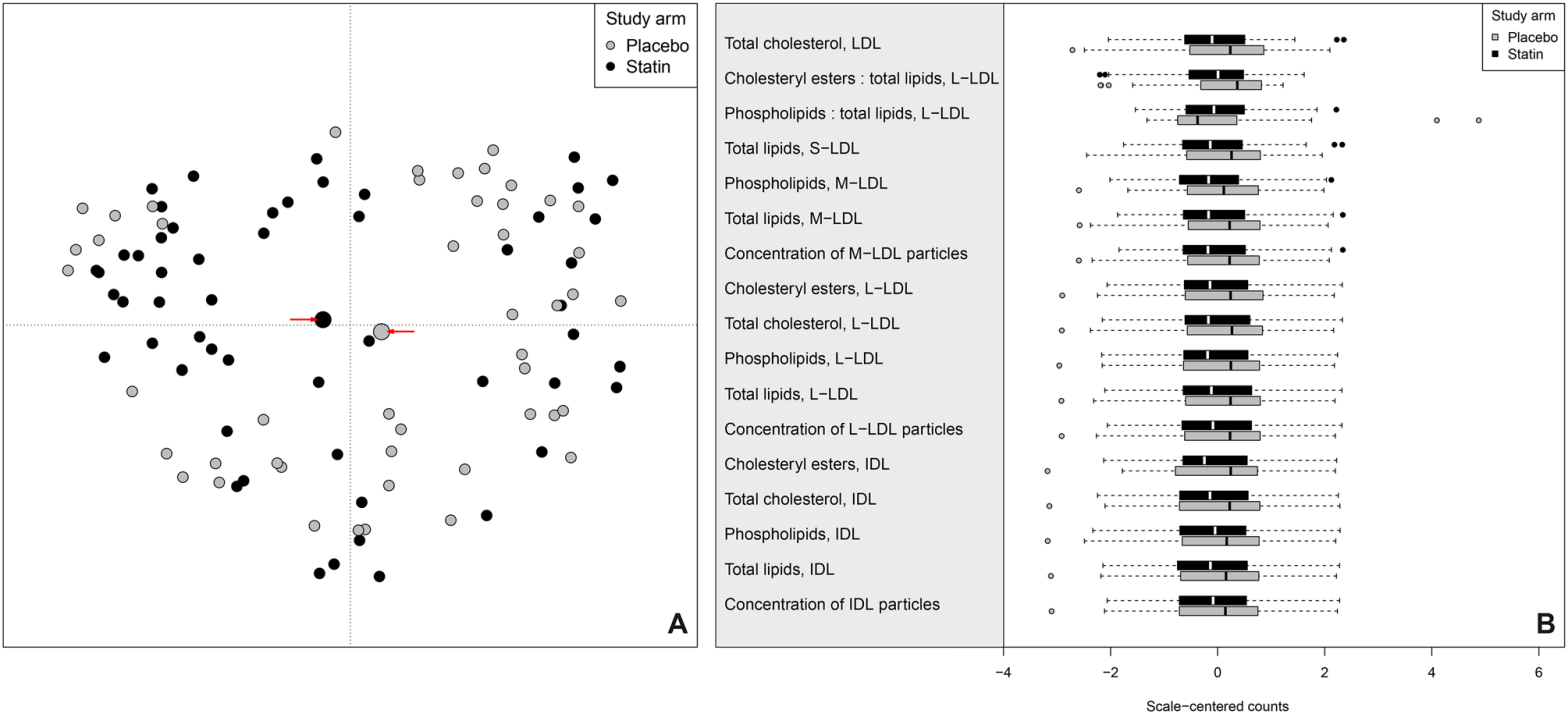
Baseline serum lipopoprotein subfraction lipid concentrations. (**A**) Proximity plot with baseline serum lipoprotein lipid patterns used as classifier results a random pattern. Random Forest classification proximity plot dimensions have no trivial interpretation therefore the names of the axes are disabled. The grey large point is the centroid (highlighted with arrows) for the placebo group and black for the statin group; the further away the centroids are, the more distinct the two classes are with respect to each other. (**B**) The baseline serum lipoprotein profiles shows equal distribution between the study arms as the median (thick bar inside a box) and dispersion (width of the box and the whiskers) are nearly overlapping. The outlying points outside the whisker region are defined as 1.5 interquartile range from either the bottom or the top quartile. The displayed metabolites are selected according to top-performing RFC classifiers in the serum metabolome after the intervention.

The clinical and other patient characteristics according to study arms are shown in Table 1. The baseline serum metabolome-based lipid and lipoprotein lipid profiles are random between the study arms (Fig. 2A) and there is no statistically significant difference in the median lipid levels (Fig. 2B). Effect size as median lipid levels difference between the treatment arms weighted by intervention duration or Gleason grade was neglectable and close to zero (Supplementary file 3, Table 1, 2 and 4). The median classification error is 46.6% (95% estimated confidence interval 40.7–51.4%). The poor classification performance indicates that there is no association between baseline serum lipidome and randomization of the study arms, as should be expected. Baseline lipids did not demonstrate statistically significant median difference between the study arms after Benjamini–Hochberg adjustment (all *p*-values are close to 1). Thus, the randomization of the treatment arms was conducted successfully.

**Figure 3:**
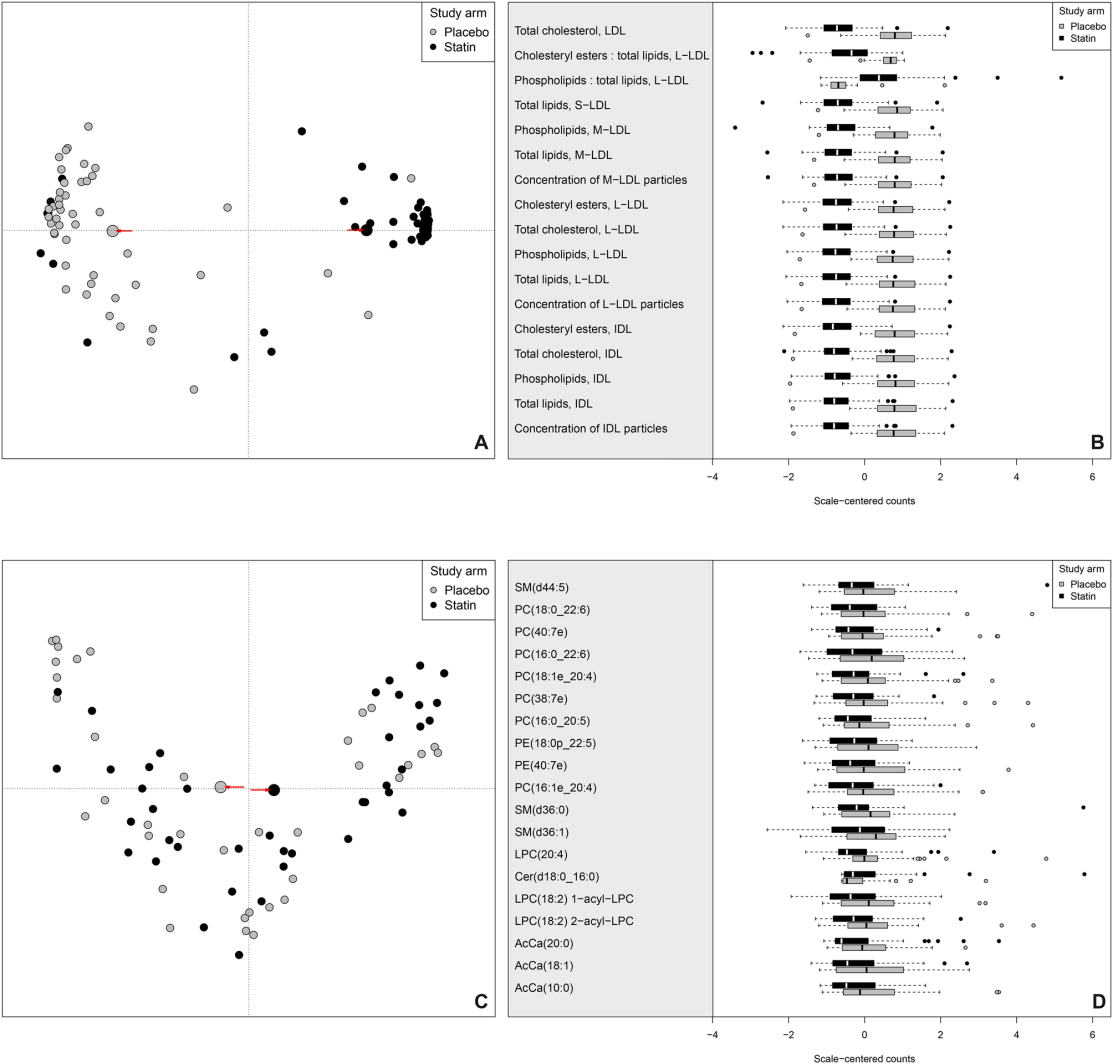
Serum lipoprotein lipid concentrations and intraprostatic tissue lipidome after atorvastatin intervention*.* (**A**) Proximity plot with serum lipidome after the intervention used as classifier shows clear pattern and distinct centroids (grey and black large points highlighted with arrows), indicating about outstanding classification performance. (**B**) The serum metabolite profile demonstrates differing distributions between the study arms; the median lipoprotein levels are clearly different whereas the dispersions are nearly equal. Note that, one extreme outlying point at − 5.62 in the cholesteryl esters: total lipids, L-LDL statin arm is not visualized to retain visual clarity of the rest of the boxplot. (**C**) The proximity plot with intraprostatic lipidome used as classifier does not result a clear pattern; only at the very tips of the arms few points are clearly clustered among both study arms. (**D**) Intraprostatic lipid level distributions shows slightly differing median lipid levels where statin arm lipid levels are consistently lower than placebo arm lipid levels, except Cer(d18:0 16:0). Moreover, the lipid levels display high dispersion in both arms (wide box, even wider whiskers and far outliers outside 1.5 interquartile range).

After the atorvastatin intervention, the study arms display clearly distinct serum lipidomic profiles (Fig. 3A). The median classification error is 11.6% (95% estimated confidence interval 10.6–13.6%). The low classification error indicates that there is a clear association between the post intervention serum lipoprotein lipid content and atorvastatin use. The best performing classifier lipids, according to the random forest, are systematically lower among the atorvastatin group with the exception of phospholipids to total lipids ratio in large LDL, which is higher in the statin group (Fig. 3B). Furthermore, the best classifying lipids display statistically significant median difference between the study arms, according to Wilcoxon rank-sum test (all Benjamini–Hochberg adjusted *p*-values smaller than 1 × 10^–10^). The effect size as median difference between the treatment arms weighted by intervention duration or Gleason grade was clearly up from before the intervention lipid level difference (Supplementary file 3, Tables 1, 2 and 4). This is a clear evidence that atorvastatin intervention resulted as a systematic shift in serum lipid and lipoprotein lipid profiles among prostate cancer patients.

The intraprostatic LC–MS/MS based lipidome profile displays slim difference after the statin intervention between the study arms (Fig. 3C). The median classification error is 44.7% (95% estimated confidence interval 40.7–50%). Such high classification performance is considered as poor, and at maximum suggests a slight association between intraprostatic lipidome and atorvastatin intervention. However, statin arm median classification error is 37.5% (95% estimated confidence interval 32.5–42.5%) whereas placebo arm median classification error is 52.7% (95% estimated confidence interval 47.2–61.1%). This indicates that the intraprostatic lipidome classifies the statin arm significantly better compared to the placebo arm. The difference likely stems from the harmonizing effect of atorvastatin use suggesting that the intraprostatic lipidome has indeed shifted due to atorvastatin use. Intraprostatic tissue lipids do not show statistically significant median difference between the study arms anymore after *p*-value adjustment. However, there is a visible difference in the lipidome levels between the study arms when the distributions are displayed side-by-side. See, for example, the differing levels of LPC(20:4) between the treatment arms (Fig. 3D). The median difference in the intraprostatic tissue lipidome between the treatment arms weighted by intervention duration or Gleason grade is not as pronounced as in the serum lipoprotein lipid concentrations but is still visible (Supplementary file 3, Tables 1, 3, and 5).

### Influence of tumor Gleason grade and duration of statin use to intraprostatic tissue lipidome shift

**Figure 4:**
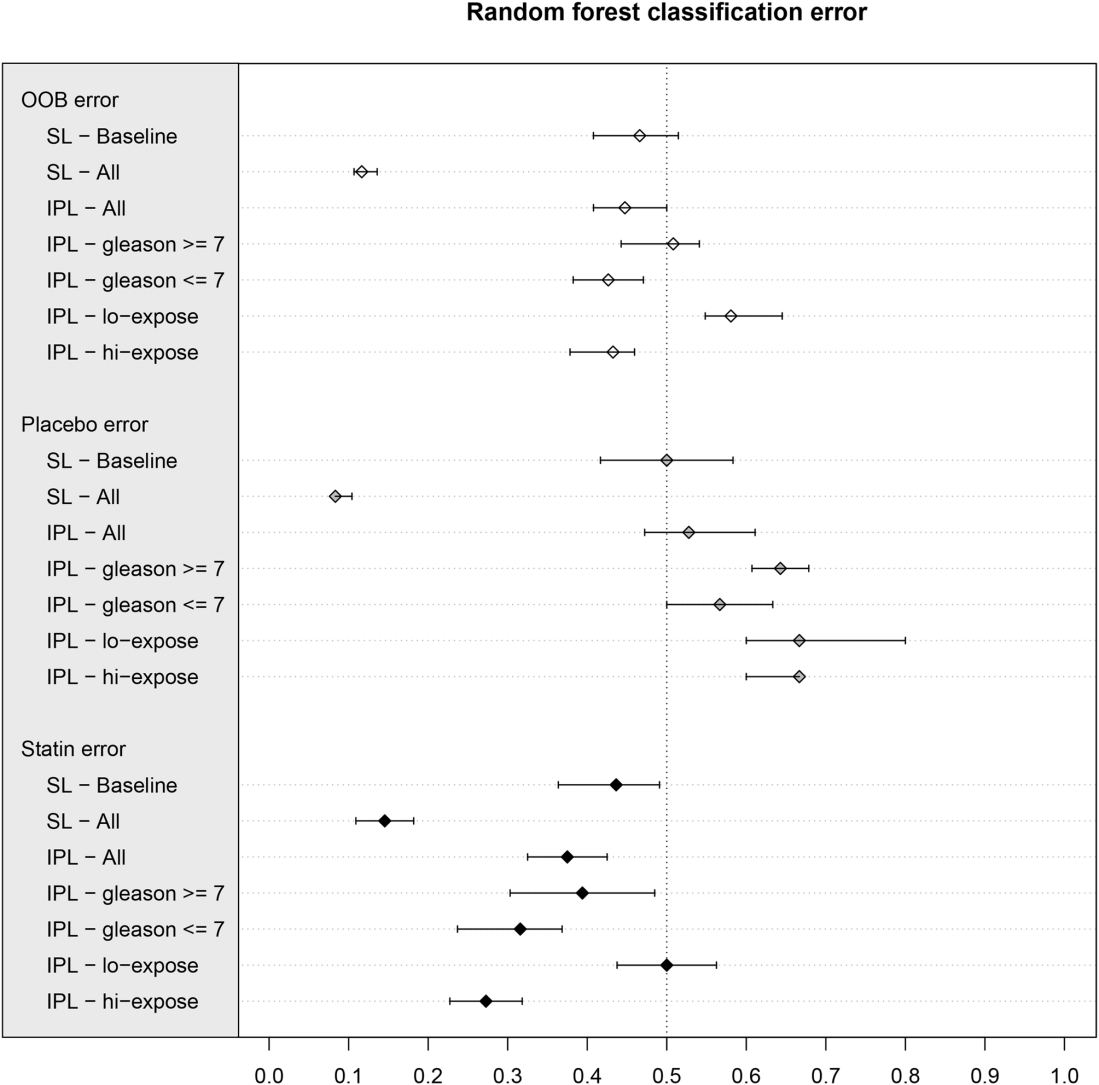
Summary of all RFC model results depicted as a forest plot. The diamonds correspond to the point estimates (median) of each model: white for out-of-bag classification error; grey for placebo group classification error; black for statin group classification error, whereas the bars represent the estimated 95% confidence interval of the corresponding classification error. The vertical dashed line represents the 50% classification error, which is considered as random. OOB error and estimated 95% confidence interval below 50% indicates that the classifiers perform better than random. The serum lipid and lipoprotein lipids (SL) after the statin intervention performs extremely well as the classifier, whereas the intraprostatic tissue lipidome (IPL) performs barely below the 50% line. Remarkably, in each intraprostatic lipidome model, the statin arm in much better classified in comparison to the placebo arm. Intraprostatic lipidome (IPL) does not discriminate the study arms among men with Gleason 7-and-above, whereas it does separate the study arms among men with Gleason 7-and-below. The intraprostatic lipidome (IPL) cannot make a distinction between the study arms in below-27-days (lo-expose) statin use sub-population, while it does discriminate the study arms among men who used stains more than 27 days (hi-expose).

Men having tumor Gleason score 7 or below displays better median classification error, 42.6% (95% estimated confidence interval 38.2–47.1%), in comparison to sub-population of men with Gleason score 7 or above showing median classification error of 50.8% (95% estimated confidence interval 44.3–54.1%) (Fig. 4). The 8% difference in the median classification errors might indicate that there is an association between intraprostatic lipidome and atorvastatin use in the Gleason 7-and-below sub-population, while there is no such association in the Gleason 7-and-above sub-population. In the Gleason 7-and-below sub-population, the placebo group median classification error is 56.6% (95% estimated confidence interval 50–63.3%) whereas the statin group median classification error is significantly lower at 31.5% (95% estimated confidence interval 23.6–36.8%). This indicates that the statin arm might be better characterized by the intraprostatic lipidome than the placebo arm. The effect could, again, be due to statin group having a harmonizing factor, statin use, which can be expected to similarize the group with respect to the lipidome, whereas the placebo group should display randomly distributed lipidome profiles at all times. There is no significant class imbalance between the study arms in Gleason score sub-population models (Gleason 7-and-below: placebo n = 30, statin n = 38 and Gleason 7-and-above: placebo n = 28, statin n = 33) to induce bias to the RFC model.

Men who used medication over 27 days (hi-expose) prior to radical prostatectomy show significantly lower median classification error of 43.2% (95% estimated confidence interval 37.8–45.9%), when compared to median classification error of 58.1% (95% estimated confidence interval 54.8–64.5%) for men who used medication for less than 27 days (lo-expose) (Fig. 4). This suggests that the longer statin exposure time results as more pronounced intraprostatic lipidome shift, compared to shorter exposure times. Moreover, the hi-expose statin arm median classification error is 27.2% (95% estimated confidence interval 22.7–31.8%) and is significantly better in comparison to the corresponding placebo users displaying median classification error of 66.6% (95% estimated confidence interval 60–66.6%). The difference is likely due to the harmonizing effect of statin use and the neutral effect of placebo use. The classes are well balanced in this sub-population analysis (hi-expose: placebo n = 15, statin n = 22 and lo-expose: placebo n = 15, statin n = 16) making the model unlikely to demonstrate class imbalance induced bias.

### Sensitivity analyses

There were some baseline differences in smoking, hypertension, and diabetes status between the treatment arms. However, the serum lipoprotein lipid concentrations before or after the intervention did not significantly differ by these baseline variables (Supplementary fiile 3, Table 6). One intraprostatic lipid PC(16:0_20:5) displayed difference (unadjusted *p*-value of Wilcoxon rank-sum test 0.004) by smoking status; however, the association can be due to change due to multiple hypothesis testing (Supplementary file 3, Table 7). Including smoking, hypertension, and diabetes status to the RFC models did not decrease the classification error compared to model without them (Supplementary file 3, Table 8). Moreover, these background covariates displayed as the least important variables in terms of improving the classification accuracy (Supplementary file 3, Fig. 1).

## Discussion

In this study we characterized serum lipoprotein lipid and prostate tissue lipidome profiles and found that high-dose atorvastatin intervention changes the serum lipid and lipoprotein lipid profile as should be expected. More importantly, we also observed a slight association between atorvastatin use and intraprostatic tissue lipidome shift. Moreover, the association became more pronounced among men who had used atorvastatin longer than 27 days prior surgery. This might reflect the pharmacodynamics of statins and the resulting LDL cholesterol reduction, which is observed to reach steady-state after 28 to 42 days from therapy initiation in a clinical setting^24^.

The association between statin use and intraprostatic lipidome shift were slightly clearer among men having Gleason score equal-or-less-than 7. However, the median classification error difference between the Gleason-score sub-populations is small, compared to the corresponding difference between high- and low-exposed groups. The result suggests that PrCa aggressiveness does not significantly, if at all, modify the association between statin use and intraprostatic lipidome. Moreover, the post hoc analysis on association between statin use and Gleason score by intervention time is hypothesis generating therefore the results should be interpreted cautiously.

Solid tumor cancer, such as prostate cancer, typically demonstrate hostile hypoxic microenvironment and low nutrient supply^25,26^. The cancer cell needs fatty acids for growth and the fatty acid supply is normally facilitated by pathways requiring oxygen^27^. During hypoxia, the fatty acid production by oxygen-consuming reactions is limited; however, in vitro hypoxic cells begin to directly harvest and increase the intake of unsaturated lipids from their surroundings, including from the serum^28^. Specifically, lysolipids have been found to support growth in Ras-driven cells^28^. Concordantly, our analysis shows an association between the decrease of unsaturated lysophosphatidylcholines LPC(20:4) and LPC(18:2) in the intraprostatic tissue and atorvastatin intervention. Therefore, suggesting that atorvastatin inhibits a central adaptation mechanism to hypoxia in prostate cancer.

Lipids have multiple important roles in PrCa cells, including energy storage and production, structural components in the membranes, signaling molecules, post-translational protein modifications, and as a substrate for steroid production^8^. Extracellular vesicles, released from cells into the extracellular environment, are involved in cell-to-cell interaction, affecting cellular functions. Moreover, extracellular vesicles promote tumor progression in PrCa^29^. The lipidome of extracellular vesicles is shown to be different between PrCa cells and normal prostate cells^13^. Since lipids have multiple important regulatory functions both, intra- and extracellularly, it is reasonable to assume that the changes in intraprostatic lipidome reflect proliferation and progression of cancer. This trial previously demonstrated that atorvastatin intervention lowers PrCa proliferation compared to placebo, the effect getting stronger along with duration of exposure^16^. After minimum exposure of 27 days, both the difference in Ki-67 and intraprostatic lipidome, are clear.

Importance in cholesterol and lipid metabolism in PrCa has been demonstrated by studies reporting increased de novo cholesterol synthesis^8^ and upregulated intake of fatty acids^9^ in PrCa. HMG-CoA reductase is shown to be expressed in cancer and normal prostate cells^30^, suggesting active cholesterol biosynthesis in both cell lines. However, the cholesterol homeostasis of PrCa cells appears to be dysregulated^31^. With the lack of major cholesterol exporter ABCA1 in PrCa cells^30^, we deduce that the lipidome profiles differ between PrCa- and normal prostate cells. Burch et al. have studied prostate lipidome in normal cells, primary adenocarcinoma, and cells derived from metastasis sites in vitro^32^. They found differences in phospholipid profiles between the cell groups. In our study, prostate tissue samples were taken from macroscopically cancer-free tissue area. Therefore, the lipidome in these samples does not necessarily represent the lipidome of PrCa cells but more likely the lipidome of presumably normal prostate cells of a PrCa patient.

We suggest that there are two possibilities how atorvastatin therapy changes the prostatic lipidome. Local inhibition of HMG-CoA reductase in the prostate may affect local lipidome directly by reducing local cholesterol synthesis. PrCa cells have been shown to have high expression of HMG-CoA reductase, which has also been shown to respond to statin exposure^30^. An other possibility is that serum lipids may accumulate in the prostate. Therefore, during a longer atorvastatin exposure, the lipidome of the prostate may change as a reflection of a long-term effect on the serum lipidome.

The local inhibition of the HMG-CoA reductase is more likely to explain the difference of prostate lipidome between study arms, since the atorvastatin exposure may have been too short (median: 27 days) to affect lipid accumulation in the prostate. We have previously demonstrated the ability of atorvastatin to diffuse through cell membranes as demonstrated by measurable intraprostatic atorvastatin concentrations^33^. The finding supports our hypothesis of local inhibition. It is also possible that both, reduced local synthesis and inhibition of lipid accumulation may have a role in changing the lipidome in the prostate. A further study could employ a genome wide association study including single nucleotide polymorphisms involved in cholesterol metabolizing enzymes and their association on PrCa risk and survival.

One of the strengths of this study is the precise lipidome profiles obtained by using mass spectrometry and liquid chromatography. Randomized placebo-controlled (RCT) study design enabled us to assess which changes/differences were effects of atorvastatin therapy, as known and unknown confounding factors are randomly distributed between the study arms in the trial population. This is a subset of an RCT thus due to coincidence there might be some differences in baseline covariates such as diabetes, smoking, and hypertension. Nevertheless, these differences did not affect lipid levels therefore they were not confounding our results. To our knowledge, this is the first study that examines effects of statins on both detailed metabonomic and lipidome profiles in the serum and the prostate tissue in PrCa patients in randomized study setting. This study supports the notion of direct effect of atorvastatin in the prostate.

One of the limitations of this study is that the intraprostatic lipidome profile is obtained only after the intervention. This prevents us from evaluating the change in the lipidome profile from the level before the intervention. Furthermore, the atorvastatin intervention was relatively short as typically statins are used for years.

## Conclusion

Short-term high-dose atorvastatin intervention causes a clear change in serum lipid and lipoprotein lipids and a moderate change in the intraprostatic tissue lipidome when compared to placebo, among men with PrCa. Therefore, atorvastatin therapy has systemic response and local effects in the prostate tissue. This is the first time that using both NMR metabolomic and LC–MS/MS based lipidomic profiles are measured with this resolution both, from the serum and the prostate tissue, in vivo human clinical trial. We suggest, that the observed survival benefit of statins is partly mediated by systemic and tissue level lipidomic change. Remarkably, lipids that are important for adaptation to hypoxic microenvironment were downshifted in atorvastatin arm, suggesting that statins may increase cancer cell vulnerability.

## Supplementary information

Supplementary file 1.

Supplementary file 2.

Supplementary file 3.

## Abbreviations

PrCa: Prostate cancer
RFC: Random forest classification
SL: Serum metabolome based lipidome
IPL: Intraprostatic Lipidome
L-LDL: Large LDL
M-LDL: Medium LDL
S-LDL: Small LDL
