# Supplementary material for: Circulatory and prostatic tissue lipidomic profiles shifts after high-dose atorvastatin use in men with prostate cancer
Source: Sci Rep. 2020 Jul 21;10:12016. doi: 10.1038/s41598-020-68868-5 (PMC7374714; doi:10.1038/s41598-020-68868-5)
Supplement: Supplementary file 2 [file 41598_2020_68868_MOESM2_ESM.docx]

**Sample preparation and extraction**

*Sample prep*

Tissue samples were homogenized by freezing tissue with liquid nitrogen and grinding it with mortar. Ground tissue was dissolved in 0,9% NaCl solution. The concentration of homogenates was 100 mg of tissue in one milliliter of 0,09 NaCl solution. Homogenates were frozen and stored at -80°C.

*Extraction*

First 30 microliters of melted prostate homogenate was placed into a clean 2ml Eppendorf tube and 5 microliters of lipid standard (SPLASH Lipidomics®, Avanti Polar Lipids) was added to the sample mixture. 225 microliters of cold methanol with 1 mM BHT was added to 30 microliters of homogenate mixture. Then 750 microliters of cold methyl-tert-butyl ether were added and the mixture was incubated in a shaker at room temperature for one hour. After incubation 188 microliters of water was added into the mixture, hence forming two separate phases. Then the mixture was incubated at room temperature for ten minutes. The mixture was vortexed twice during that time.

After incubation, the mixture was centrifuged for ten minutes at 1500g at +5 °C. Upon centrifugation, the lower water layer was frozen with dry ice and the upper organic phase was poured into a clean 2 ml Eppendorf tube. The lower water phase was reextracted with 1500 microliters of a mixture containing MTBE:methanol:water (10:3:2,5) and centrifuged. The lower phase was again frozen with dry ice and organic layers were combined and dried with a nitrogen evaporator (Organomation Associates Inc, N-EVAP^TM^ 112).

**LC-MS analysis**

*UPLC separation*

Separation of lipids was accomplished by using a Poroshell 120 EC-C18 column (2.1 x 100mm, 2.7 μm, Agilent) using Vanquish UPLC system (Thermo Scientific), using a flow rate of 0.4 μL/min and injection volume of 2 μL. The temperature of column was set on 40 °C. Mobile phase consisted of a combination on eluent A (60:40 acetonitrile: water + 10mM ammonium formate + 0,1% formic acid) and eluent B ( 85:10:5, isopropanol: Acetonitrile: water + 10mM ammonium formate + 0,1 % formic acid). The gradient of B was raised from 5% to 100% within 0-19 minutes of run, held at 100% from 19 to 21 minutes and then lowered from 100% to 5% between 21 and 21.1 minute. 5% of B was held till the end of run at 25 minutes.

*Positive mode*

Q-Exactive Focus orbitrap mass spectrometer (Thermo Scientific) was combined with heat electrospray ionization (HESI) and operated in full scan mode. MS/MS data was collected from a couple of quality control analyses at the beginning and end of the run to obtain fragmentation spectra for identification of lipids. The flow rates of auxiliary gas, sheet gas and sweep gas were set to 10, 40 and 2 (arbitrary units). Spray voltage was set on 3500 volts under a positive mode, S-lens RF level at 55 and auxiliary gas heater temperature at 330 °C. The automatic gain control target was set on 5e5 with maximum injection time of 50 ms. Scan range was from 240-1650 m/z with the resolution on 70000 (FWHM 200 m/z)

*Negative mode*

Separation was implemented as done on positive side. Only quality control analyses were done in Full scan MS/MS mode to gain spectrum identification about the fatty acid composition of lipids. Full scan MS/MS analysis was conducted with Q-exactive Focus MS and Vanquish UHPLC system. Spray voltage was set on 3000 V, auxiliary gas flow rate at 10, sheet gas at 40 and sweep gas at 2. S-lens RF level was set on 55, auxiliary gas heater and capillary temperatures at 350 °C and 330 °C. Automatic gain control target and maximum injection time were set as were on positive side.

***Data processing***

Data was processed using two computer programs: Compound discoverer 2.1 (Thermo Fisher Scientific) and LipidSearch (Thermo Fisher Scientific). Data acquisition was done with Thermo Xcalibur software. Compound discoverer was used to detect compounds and identify differences between samples. Specimen were sorted into two groups: sample group in which sample donors were given atorvastatin, and control group consisting samples from participants who were given placebo. The program performed retention time alignment, unknown compound detection and compound grouping across all samples. The program calculated differential analysis (t-test and ANOVA), determines p-value (t-test), adjusted p-value for false discovery rate, ratios (Sample /control), fold change, CV (coefficient of variation). The program tried to identify compounds with its own MzVault spectral library, but the library wasn’t comprehensive enough to identify lipids from samples, so LipidSearch program was used to identify lipids.

LipidSearch analysis was done from two quality control samples, one from positive and one from negative side whereof MS/MS data was acquired. LipidSearch identified lipids by detecting the peak, calculating mass and comparing information to its own database containing more than 200 000 theoretical m/z values for lipid ions and their theoretical fragment ions. The fragmentations patterns are theoretically calculated and improved with experts’ knowledge of experimental fragmentation of lipids. For identification on lipid molecules found by Compound Discoverer the alignment of two LipidSearch analyses was used. Lipids were identified by comparing molecular weight and retention time of compound obtained with Compound Discoverer to molecular weight and retention time from LipidSearch.
