# Supplementary material for: Circulatory and prostatic tissue lipidomic profiles shifts after high-dose atorvastatin use in men with prostate cancer
Source: Sci Rep. 2020 Jul 21;10:12016. doi: 10.1038/s41598-020-68868-5 (PMC7374714; doi:10.1038/s41598-020-68868-5)
Supplement: Supplementary file 3 [file 41598_2020_68868_MOESM3_ESM.docx]

Table 1. Serum lipoprotein lipid concentration before and after statin intervention, and intraprostatic tissue lipid peak area, median and interquartile range (IQR) for placebo and statin arms separately.

| *Serum lipoprotein lipid before, mmol / l* | *Placebo, median (IQR)* | *Statin, median (IQR)* |
| --- | --- | --- |
| Total cholesterol, LDL | 1.68 (0.52) | 1.55 (0.43) |
| Cholesteryl esters : total lipids, L-LDL | 48.77 (2.47) | 47.97 (2.26) |
| Phospholipids : total lipids, L-LDL | 25.22 (1.4) | 25.61 (1.41) |
| Total lipids, S-LDL | 0.48 (0.13) | 0.45 (0.11) |
| Phospholipids, M-LDL | 0.2 (0.04) | 0.19 (0.03) |
| Total lipids, M-LDL | 0.75 (0.21) | 0.69 (0.18) |
| Concentration of M-LDL particles | 0 (0) | 0 (0) |
| Cholesteryl esters, L-LDL | 0.63 (0.2) | 0.57 (0.17) |
| Total cholesterol, L-LDL | 0.88 (0.26) | 0.79 (0.23) |
| Phospholipids, L-LDL | 0.33 (0.07) | 0.3 (0.06) |
| Total lipids, L-LDL | 1.29 (0.35) | 1.2 (0.32) |
| Concentration of L-LDL particles | 0 (0) | 0 (0) |
| Cholesteryl esters, IDL | 0.49 (0.14) | 0.45 (0.12) |
| Total cholesterol, IDL | 0.68 (0.21) | 0.63 (0.18) |
| Phospholipids, IDL | 0.29 (0.08) | 0.28 (0.07) |
| Total lipids, IDL | 1.07 (0.29) | 1.01 (0.26) |
| Concentration of IDL particles | 0 (0) | 0 (0) |
|  |  |  |
| *Serum lipoprotein lipid after, mmol / l* | *Placebo, median (IQR)* | *Statin, median (IQR)* |
| Total cholesterol, LDL | 1.63 (0.41) | 0.84 (0.39) |
| Cholesteryl esters : total lipids, L-LDL | 48.55 (2.1) | 42.1 (5.82) |
| Phospholipids : total lipids, L-LDL | 25.41 (1.14) | 28.65 (2.92) |
| Total lipids, S-LDL | 0.48 (0.11) | 0.27 (0.1) |
| Phospholipids, M-LDL | 0.2 (0.04) | 0.13 (0.04) |
| Total lipids, M-LDL | 0.74 (0.17) | 0.4 (0.15) |
| Concentration of M-LDL particles | 0.000144 (0.000034) | 0.000078 (0.000031) |
| Cholesteryl esters, L-LDL | 0.6 (0.17) | 0.3 (0.15) |
| Total cholesterol, L-LDL | 0.84 (0.22) | 0.44 (0.19) |
| Phospholipids, L-LDL | 0.31 (0.07) | 0.2 (0.05) |
| Total lipids, L-LDL | 1.23 (0.32) | 0.71 (0.25) |
| Concentration of L-LDL particles | 0.000172 (0.000044) | 0.000099 (0.000035) |
| Cholesteryl esters, IDL | 0.47 (0.14) | 0.25 (0.1) |
| Total cholesterol, IDL | 0.65 (0.18) | 0.35 (0.12) |
| Phospholipids, IDL | 0.29 (0.07) | 0.17 (0.05) |
| Total lipids, IDL | 1.05 (0.27) | 0.61 (0.18) |
| Concentration of IDL particles | 0.000103 (0.000025) | 0.000061 (0.000017) |
|  |  |  |
| *Intraprostatic tissue lipid, peak areas* | *Placebo, median (IQR)* | *Statin, median (IQR)* |
| SM(d44:5) | 737633.21 (560171.88) | 599449.17 (404294.16) |
| PC(18:0_22:6) | 1916534.74 (1398771.87) | 1470705.35 (1456201.42) |
| PC(40:7e) | 187157.44 (136970.92) | 139880.33 (121445.97) |
| PC(16:0_22:6) | 6110786.66 (4740701.81) | 4629668.83 (4138550.17) |
| PC(18:1e_20:4) | 840836.13 (597694.08) | 643860.09 (503600.23) |
| PC(38:7e) | 413484.62 (265876.72) | 344132.48 (255081.57) |
| PC(16:0_20:5) | 1140986.2 (947022.76) | 894150.79 (754761.96) |
| PE(18:0p_22:5) | 756141.99 (633168.77) | 600891.29 (496552.54) |
| PE(40:7e) | 1275417.52 (1400999.81) | 994676.7 (882873.37) |
| PC(16:1e_20:4) | 3571225.6 (2274657.11) | 3037269.87 (2244196.43) |
| SM(d36:0) | 297016.61 (198865.08) | 238503.11 (124013.19) |
| SM(d36:1) | 5413575.34 (2228033) | 4682616.06 (2358117.81) |
| LPC(20:4) | 34649.8 (11329.26) | 26362.02 (12667.51) |
| Cer(d18:0_16:0) | 19507.9 (53482.31) | 37065.97 (89242.25) |
| LPC(18:2) 1-acyl-LPC | 67913.52 (41337.91) | 53076.94 (35143.56) |
| LPC(18:2) 2-acyl-LPC | 50359.38 (26649.42) | 41609.98 (25429.82) |
| AcCa(20:0) | 82620.89 (81285.92) | 38349.27 (65054.43) |
| AcCa(18:1) | 113906.43 (131550.85) | 76170.88 (78886.12) |
| AcCa(10:0) | 12832.36 (14707.31) | 8811.26 (12332.47) |

Table 2. Statin intervention effect size presented as median serum lipoprotein lipid levels weighted by intervention duration. The median difference (placebo – statin) between the treatment arms (Diff.) after the statin intervention is more pronounced compared to serum lipoprotein lipid level difference before the intervention.

|  | **Before intervention** | | | **After intervention** | | |
| --- | --- | --- | --- | --- | --- | --- |
| *Serum lipoprotein lipid after, mmol / l* | Placebo | Statin | Diff. | Placebo | Statin | Diff. |
| Total cholesterol, LDL | 1.68 | 1.58 | 0.10 | 1.61 | 0.81 | 0.79 |
| Cholesteryl esters : total lipids, L-LDL | 48.74 | 48.00 | 0.74 | 48.47 | 41.81 | 6.66 |
| Phospholipids : total lipids, L-LDL | 25.24 | 25.59 | -0.35 | 25.43 | 28.89 | -3.46 |
| Total lipids, S-LDL | 0.48 | 0.45 | 0.03 | 0.48 | 0.26 | 0.21 |
| Phospholipids, M-LDL | 0.20 | 0.19 | 0.01 | 0.20 | 0.13 | 0.07 |
| Total lipids, M-LDL | 0.75 | 0.71 | 0.04 | 0.73 | 0.40 | 0.33 |
| Concentration of M-LDL particles | 0.00015 | 0.00014 | 0.000008 | 0.00014 | 0.00008 | 0.00006 |
| Cholesteryl esters, L-LDL | 0.62 | 0.59 | 0.04 | 0.59 | 0.30 | 0.29 |
| Total cholesterol, L-LDL | 0.88 | 0.83 | 0.05 | 0.81 | 0.44 | 0.37 |
| Phospholipids, L-LDL | 0.32 | 0.31 | 0.01 | 0.31 | 0.20 | 0.11 |
| Total lipids, L-LDL | 1.29 | 1.24 | 0.05 | 1.21 | 0.70 | 0.51 |
| Concentration of L-LDL particles | 0.00018 | 0.00017 | 0.00001 | 0.00017 | 0.00010 | 0.00007 |
| Cholesteryl esters, IDL | 0.48 | 0.46 | 0.02 | 0.47 | 0.24 | 0.23 |
| Total cholesterol, IDL | 0.68 | 0.66 | 0.01 | 0.65 | 0.35 | 0.30 |
| Phospholipids, IDL | 0.29 | 0.29 | 0.00 | 0.28 | 0.17 | 0.11 |
| Total lipids, IDL | 1.07 | 1.06 | 0.01 | 1.04 | 0.60 | 0.44 |
| Concentration of IDL particles | 0.00011 | 0.00010 | 0.000001 | 0.00010 | 0.00006 | 0.00004 |

Table 3. Statin intervention effect size on intraprostatic tissue lipidome presented as follow-up time weighted median. There is clear difference (placebo – statin) in the intraprostatic tissue lipidome between the treatment arms.

| *Intraprostatic tissue lipid, peak areas* | Placebo | Statin | Difference |
| --- | --- | --- | --- |
| SM(d44:5) | 818564 | 570258 | 248306 |
| PC(18:0_22:6) | 2059109 | 1633856 | 425253 |
| PC(40:7e) | 209254 | 142137 | 67116 |
| PC(16:0_22:6) | 6528529 | 4757097 | 1771432 |
| PC(18:1e_20:4) | 863505 | 674685 | 188820 |
| PC(38:7e) | 426731 | 354816 | 71915 |
| PC(16:0_20:5) | 1068416 | 955659 | 112758 |
| PE(18:0p_22:5) | 869267 | 621168 | 248100 |
| PE(40:7e) | 1516821 | 1005077 | 511744 |
| PC(16:1e_20:4) | 3944559 | 3193153 | 751406 |
| SM(d36:0) | 315282 | 218157 | 97125 |
| SM(d36:1) | 5676750 | 4680970 | 995780 |
| LPC(20:4) | 34112 | 25221 | 8891 |
| Cer(d18:0_16:0) | 22451 | 37767 | -15316 |
| LPC(18:2).1 | 68430 | 54037 | 14392 |
| LPC(18:2) | 50364 | 42631 | 7733 |
| AcCa(20:0) | 87156 | 39208 | 47948 |
| AcCa(18:1) | 112234 | 81570 | 30664 |
| AcCa(10:0) | 11508 | 10529 | 979 |

Table 4. Statin intervention effect size presented as median serum lipoprotein lipid levels weighted by Gleason grade. The median difference (placebo – statin) between the treatment arms (Diff.) after the statin intervention is more pronounced compared to serum lipoprotein lipid level difference before the intervention.

|  | **Before intervention** | | | **After intervention** | | |
| --- | --- | --- | --- | --- | --- | --- |
| *Serum lipoprotein lipid after, mmol / l* | Placebo | Statin | Diff. | Placebo | Statin | Diff. |
| Total cholesterol, LDL | 1.68 | 1.55 | 0.13 | 1.63 | 0.84 | 0.79 |
| Cholesteryl esters : total lipids, L-LDL | 48.81 | 47.97 | 0.84 | 48.60 | 42.07 | 6.53 |
| Phospholipids : total lipids, L-LDL | 25.17 | 25.61 | -0.44 | 25.39 | 28.65 | -3.26 |
| Total lipids, S-LDL | 0.48 | 0.45 | 0.04 | 0.48 | 0.27 | 0.21 |
| Phospholipids, M-LDL | 0.20 | 0.19 | 0.01 | 0.20 | 0.13 | 0.07 |
| Total lipids, M-LDL | 0.75 | 0.69 | 0.06 | 0.74 | 0.40 | 0.34 |
| Concentration of M-LDL particles | 0.00015 | 0.00014 | 0.00001 | 0.00014 | 0.00008 | 0.00006 |
| Cholesteryl esters, L-LDL | 0.63 | 0.57 | 0.06 | 0.60 | 0.30 | 0.30 |
| Total cholesterol, L-LDL | 0.88 | 0.79 | 0.08 | 0.84 | 0.44 | 0.39 |
| Phospholipids, L-LDL | 0.33 | 0.30 | 0.02 | 0.31 | 0.20 | 0.11 |
| Total lipids, L-LDL | 1.29 | 1.20 | 0.09 | 1.23 | 0.71 | 0.53 |
| Concentration of L-LDL particles | 0.00018 | 0.00017 | 0.00001 | 0.00017 | 0.00010 | 0.00007 |
| Cholesteryl esters, IDL | 0.49 | 0.45 | 0.05 | 0.47 | 0.25 | 0.22 |
| Total cholesterol, IDL | 0.68 | 0.63 | 0.05 | 0.65 | 0.35 | 0.30 |
| Phospholipids, IDL | 0.29 | 0.28 | 0.01 | 0.29 | 0.17 | 0.11 |
| Total lipids, IDL | 1.07 | 1.01 | 0.06 | 1.05 | 0.61 | 0.44 |
| Concentration of IDL particles | 0.00011 | 0.00010 | 0.00001 | 0.00010 | 0.00006 | 0.00004 |

Table 5. Statin intervention effect size on intraprostatic tissue lipidome presented as median weighted by Gleason grade. There is clear difference (placebo – statin) in the intraprostatic tissue lipidome between the treatment arms.

| *Intraprostatic tissue lipid, peak areas* | Placebo | Statin | Difference |
| --- | --- | --- | --- |
| SM(d44:5) | 757941 | 600666 | 157275 |
| PC(18:0_22:6) | 1925106 | 1478150 | 446956 |
| PC(40:7e) | 190749 | 140622 | 50126 |
| PC(16:0_22:6) | 6269467 | 4662749 | 1606718 |
| PC(18:1e_20:4) | 848587 | 643881 | 204707 |
| PC(38:7e) | 423292 | 351094 | 72198 |
| PC(16:0_20:5) | 1143307 | 912695 | 230612 |
| PE(18:0p_22:5) | 808191 | 605143 | 203048 |
| PE(40:7e) | 1277078 | 998275 | 278804 |
| PC(16:1e_20:4) | 3904907 | 3053311 | 851596 |
| SM(d36:0) | 300034 | 238503 | 61531 |
| SM(d36:1) | 5435085 | 4680062 | 755022 |
| LPC(20:4) | 34527 | 26397 | 8131 |
| Cer(d18:0_16:0) | 19679 | 36207 | -16529 |
| LPC(18:2).1 | 68018 | 52549 | 15469 |
| LPC(18:2) | 50320 | 41547 | 8773 |
| AcCa(20:0) | 80925 | 39014 | 41911 |
| AcCa(18:1) | 113615 | 77679 | 35936 |
| AcCa(10:0) | 12492 | 8705 | 3787 |

Table 6. P-values of Wilcoxon Rank sum test between serum lipoprotein lipid concentrations for diabetes, smoking, and hypertension users / non-users. There is no statistically significant difference between any pairs at confidence level 0.05.

|  | *Unadjusted p-values of Wilcoxon Rank Sum test* | | | | | |
| --- | --- | --- | --- | --- | --- | --- |
|  | **Diabetes** | | **Smoking** | | **Hypertension** | |
| *Serum lipoprotein lipid* | Before | After | Before | After | Before | After |
| Total cholesterol, LDL | 0.210 | 0.278 | 0.608 | 0.605 | 0.656 | 0.125 |
| Cholesteryl esters : total lipids, L-LDL | 0.818 | 0.640 | 0.155 | 0.408 | 0.408 | 0.533 |
| Phospholipids : total lipids, L-LDL | 0.184 | 0.220 | 0.987 | 0.891 | 0.712 | 0.114 |
| Total lipids, S-LDL | 0.206 | 0.301 | 0.661 | 0.576 | 0.526 | 0.123 |
| Phospholipids, M-LDL | 0.383 | 0.451 | 0.750 | 0.605 | 0.978 | 0.282 |
| Total lipids, M-LDL | 0.258 | 0.319 | 0.608 | 0.571 | 0.744 | 0.153 |
| Concentration of M-LDL particles | 0.275 | 0.330 | 0.628 | 0.576 | 0.773 | 0.162 |
| Cholesteryl esters, L-LDL | 0.240 | 0.317 | 0.634 | 0.631 | 0.789 | 0.186 |
| Total cholesterol, L-LDL | 0.208 | 0.283 | 0.719 | 0.722 | 0.699 | 0.122 |
| Phospholipids, L-LDL | 0.190 | 0.278 | 0.826 | 0.763 | 0.676 | 0.149 |
| Total lipids, L-LDL | 0.216 | 0.285 | 0.801 | 0.750 | 0.694 | 0.133 |
| Concentration of L-LDL particles | 0.222 | 0.273 | 0.869 | 0.747 | 0.671 | 0.153 |
| Cholesteryl esters, IDL | 0.218 | 0.325 | 0.843 | 0.778 | 0.765 | 0.223 |
| Total cholesterol, IDL | 0.123 | 0.263 | 0.901 | 0.888 | 0.529 | 0.132 |
| Phospholipids, IDL | 0.105 | 0.172 | 0.895 | 0.882 | 0.461 | 0.067 |
| Total lipids, IDL | 0.122 | 0.244 | 0.997 | 0.927 | 0.473 | 0.123 |
| Concentration of IDL particles | 0.145 | 0.249 | 0.967 | 0.901 | 0.540 | 0.157 |

Table 7. P-values of Wilcoxon Rank sum test between intraprostatic tissue lipid concentrations for diabetes, smoking, and hypertension users / non-users. There is one statistically significant, confidence level 0.05, difference in intraprostatic lipid concentrations between non-smokers and smokers, PC(16:0_20:5), highlighted as bold font.

|  | *Unadjusted p-values of Wilcoxon Rank Sum test* | | |
| --- | --- | --- | --- |
| *Intraprostatic tissue lipid* | **Diabetes** | **Smoking** | **Hypertension** |
| SM(d44:5) | 0.578 | 0.601 | 0.515 |
| PC(18:0_22:6) | 0.578 | 0.073 | 0.730 |
| PC(40:7e) | 0.943 | 0.148 | 0.427 |
| PC(16:0_22:6) | 0.553 | 0.122 | 0.848 |
| PC(18:1e_20:4) | 0.578 | 0.455 | 0.453 |
| PC(38:7e) | 0.495 | 0.095 | 0.338 |
| PC(16:0_20:5) | 0.541 | **0.004** | 0.738 |
| PE(18:0p_22:5) | 0.429 | 0.337 | 0.487 |
| PE(40:7e) | 0.553 | 0.141 | 0.543 |
| PC(16:1e_20:4) | 0.484 | 1.000 | 0.198 |
| SM(d36:0) | 0.857 | 0.432 | 0.857 |
| SM(d36:1) | 0.332 | 0.970 | 0.618 |
| LPC(20:4) | 0.429 | 0.842 | 0.831 |
| Cer(d18:0_16:0) | 0.666 | 0.071 | 0.447 |
| LPC(18:2) 1-acyl-LPC | 0.829 | 0.092 | 0.865 |
| LPC(18:2) 2-acyl-LPC | 0.872 | 0.822 | 0.917 |
| AcCa(20:0) | 0.578 | 1.000 | 0.839 |
| AcCa(18:1) | 0.098 | 0.727 | 0.961 |
| AcCa(10:0) | 0.127 | 0.583 | 0.961 |


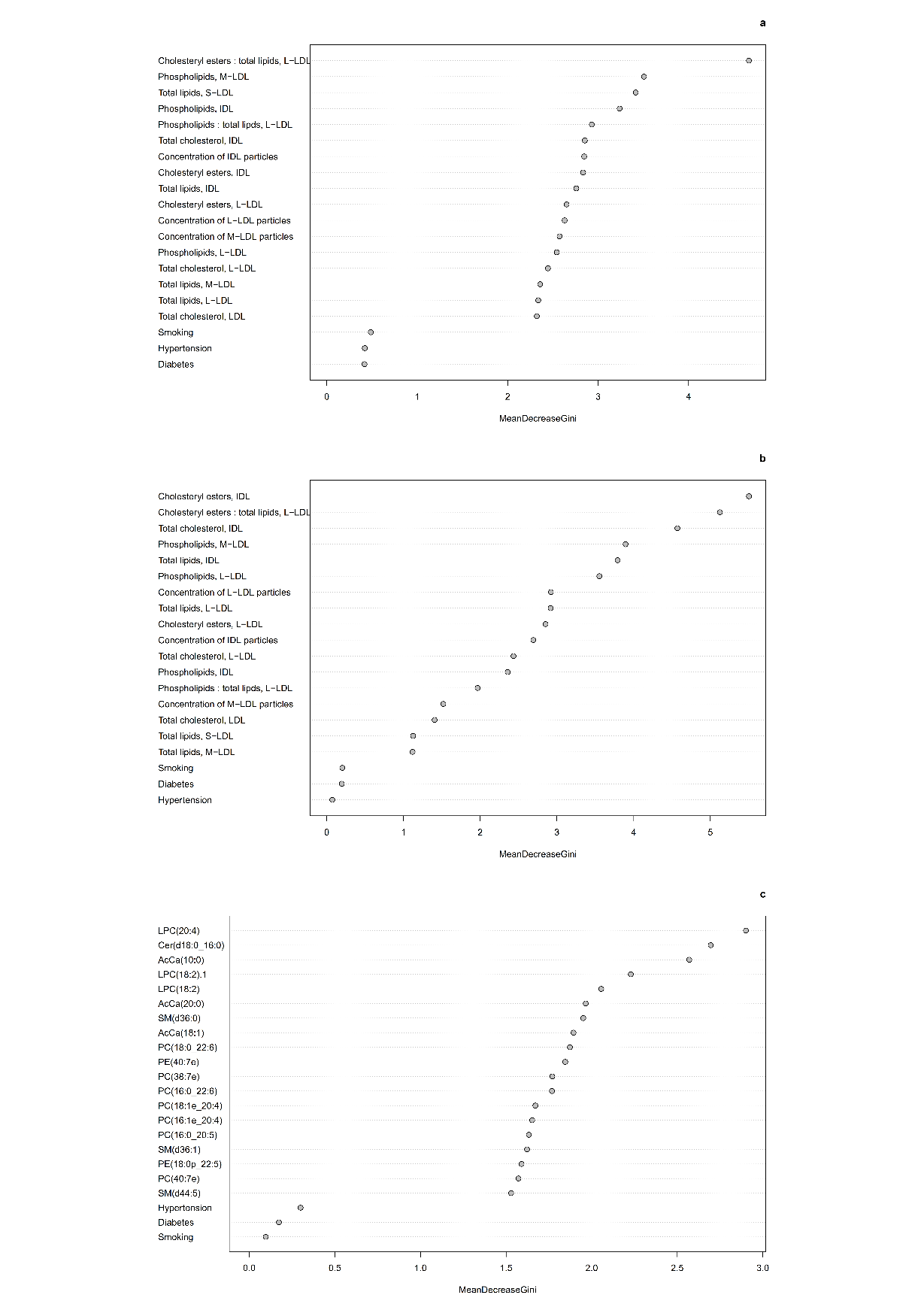


Figure 1. Variable importance in terms of improving classification accuracy of random forest classifier models adjusted for hypertension, diabetes, and smoking. a) serum lipoprotein lipids before the intervention, b) serum lipoprotein lipids after the intervention, and c) intraprostatic tissue lipid after the intervention. The three background covariates, hypertension, diabetes, and smoking does not improve the classification accuracy of the model whereas the lipid levels dominates the classification accuracy improvement.

Table 8. Random forest classification error estimate and 95% confidence interval of 1000 random forest repetitions using background covariates hypertension, diabetes, and smoking as classifiers together with the lipids.

| Model classifiers | Median Classification error (95% Confidence interval) |
| --- | --- |
| *Serum lipoprotein lipids before, hypertension, diabetes, and smoking* | 0.49 (0.45 – 0.51) |
| *Serum lipoprotein lipids after, hypertension, diabetes, and smoking* | 0.13 (0.11 – 0.15) |
| *Intraprostatic tissue lipids after, hypertension, diabetes, and smoking* | 0.46 (0.40 – 0.50) |
